# Functional connectivity and upper limb function in patients after pediatric arterial ischemic stroke with contralateral corticospinal tract wiring

**DOI:** 10.1038/s41598-021-84671-2

**Published:** 2021-03-09

**Authors:** Leonie Steiner, Stephanie Homan, Regula Everts, Andrea Federspiel, Sandeep Kamal, Juan Antonio Delgado Rodriguez, Salome Kornfeld, Nedelina Slavova, Roland Wiest, Alain Kaelin-Lang, Maja Steinlin, Sebastian Grunt

**Affiliations:** 1 Division of Neuropediatrics, Development and Rehabilitation, University Children’s Hospital, Inselspital, Freiburgstrasse 31, 3010 Bern, Switzerland; 2 Graduate School for Health Sciences, University of Bern, Bern, Switzerland; 3 Division of Systems Neuroscience, Translational Research Center, University Hospital of Psychiatry and Psychotherapy, Bern, Switzerland; 4 Psychiatric Neuroimaging Unit, Translational Research Center, University Hospital of Psychiatry and Psychotherapy, Bern, Switzerland; 5 Department of Psychiatry, Psychotherapy and Psychosomatics, University Hospital of Psychiatry, University of Zurich, Zurich, Switzerland; 6 Department of Diagnostic and Interventional Neuroradiology, Inselspital, Bern University Hospital, University of Bern, Bern, Switzerland; 7 Neurocenter of Southern Switzerland, Ente Ospedaliero Cantonale, Bellinzona, Switzerland; 8 Department of Neurology, Inselspital, Bern University Hospital, University of Bern, Bern, Switzerland; 9 Faculty of Biomedical Neurosciences, Università Della Svizzera Italiana, Lugano, Switzerland

**Keywords:** Neuroscience, Diseases of the nervous system, Stroke

## Abstract

To develop individualized motor rehabilitation, knowledge of the relationship between neuroplastic reorganization and motor recovery after pediatric arterial ischemic stroke (AIS) is crucial. Thus, we investigated functional connectivity in patients after AIS with good motor outcome and in patients with hemiparesis compared with typically developing peers. We included 18 patients (n = 9 with hemiparesis, n = 9 with good motor outcome) with pediatric AIS in the chronic phase (≥ 2 years after diagnosis, diagnosed > 16 years) and 18 peers matched by age and gender. Participants underwent a standardized motor assessment, single-pulse transcranial magnetic stimulation to determine the type of corticospinal tract wiring, and resting-state functional magnetic resonance imaging to examine motor network connectivity. Corticospinal tract wiring was contralateral in all participants. Patients with hemiparesis had lower interhemispheric connectivity strength compared with patients with good clinical outcome and peers. Patients with good clinical outcome had higher intrahemispheric connectivity strength compared with peers. Further, higher intrahemispheric connectivity was related to better motor outcome in patients. Our findings suggest that better motor outcome after pediatric AIS is related to higher motor network connectivity strength. Thus, resting-state functional connectivity might be predictive for motor recovery after pediatric AIS.

## Introduction

Arterial ischemic stroke (AIS) is a rare but devastating disease in childhood. Data from the Swiss Neuropaediatric Stroke Registry (SNPSR) indicates that 2.1/100,000 children and 13 neonates per 100,000 live births are affected each year in Switzerland^1,2^. The international comparison with the US shows comparable numbers with 2.4/100,000 children suffering an arterial or venous stroke each year^3^. However, considerable long-term sequelae such as hemiparesis can be particularly disabling^2,4–6^, leading to impaired upper limb function and limited manual dexterity, which in turn can significantly reduce the health-related quality of life of the affected children and their families^7,8^. Therefore, a better understanding of motor recovery is essential.

The severity of upper limb impairment is thought to vary according to stroke- and lesion-related characteristics such as lesion size and location, and microstructural integrity of white matter bundles^9,10^. In general, upper limb movement is facilitated by descending corticospinal pathways. Early unilateral brain injury can result in different patterns of corticospinal tract reorganization^11,12^. These include (1) ipsilateral corticospinal pathways (the representation of the paretic hand in the primary motor cortex is on the same side as the lesion), (2) contralateral corticospinal pathways (the representation of the paretic hand in the primary motor cortex is on the contralateral side to the lesion) as expected in a typically developing brain, and (3) mixed corticospinal pathways, which can be assessed with single-pulse transcranial magnetic stimulation (TMS)^13^. Previous studies suggest that patients with contralateral corticospinal tract wiring have more preserved motor function than those with mixed or ipsilateral corticospinal tract wiring^12–16^. However, these different corticospinal tract reorganization patterns and lesion-related characteristics have often limited predictive value for post-stroke motor outcome. The unexplained variability in motor outcome may be due to widespread changes in functional connectivity. Therefore, a functional connectivity-focused approach is essential to improve our knowledge of the relationship between neuroplastic reorganization and post-stroke recovery of upper limb function.

A promising tool for understanding widespread alterations in functional brain networks is resting-state functional magnetic resonance imaging (rs-fMRI)^17^. This imaging method is based on the temporal correlation of the blood-oxygen-level-dependent (BOLD) signal in distant brain regions. Studies in adults after AIS with rs-fMRI showed that interhemispheric connectivity in the motor network is crucial for motor recovery^18–23^. In particular, lower interhemispheric connectivity strength has been associated with reduced motor function^18–20,24–27^ and increases over time as a function of recovery^28,29^. Further, Mintzopoulos, et al.^30^ investigated patients with good motor recovery after AIS and found increased intrahemispheric connectivity between the primary motor cortex and supplementary motor area, most likely an adaptive compensatory process.

However, findings in adults cannot necessarily be extrapolated to children. Studies in pediatric populations with upper limb impairment after focal brain lesions are scarce. The few studies available indicate that functional connectivity of the motor network is more diffuse, leading to a potentially reduced specificity and lower network efficiency compared with functional motor network connectivity of typically developing peers^9,14,31^. Specifically, Saunders, et al.^9^ found both increased and decreased functional connectivity of the primary motor cortex to other brain regions in children with perinatal AIS compared with typically developing peers. Interestingly, functional connectivity of the primary motor cortex was unrelated to motor outcome. Thus, investigating the wider motor network and including premotor, supplementary motor, and parietal cortices may be more informative than restricting analyses to the primary motor cortices alone^9^.

Further, since previous studies showed that unilateral injury can lead to different corticospinal tract wiring patterns in early childhood, TMS is crucial to ensure that only patients with contralateral reorganization are compared with each other. Using this approach, the influence of different cortical reorganization patterns on connectivity is avoided.

Therefore, we investigated the wider motor network in patients after pediatric AIS with hemiparesis compared with patients after pediatric AIS with good motor outcome and typically developing peers. The comparison of functional connectivity between patients with poor (hemiparesis) and good motor outcome could provide valuable insights into the adaptive reorganization of functional connectivity after pediatric AIS. In accordance with the existing literature, we hypothesized that (1) patients after pediatric AIS with hemiparesis have lower interhemispheric functional connectivity in the motor network compared with patients after AIS with good motor outcome and typically developing peers, and (2) upper limb function is related to inter- and intrahemispheric network connectivity strength.

## Results

### Participant characteristics, clinical outcome, and cortical representation of hand movements

The final study population consisted of 18 patients with AIS of whom nine were diagnosed with hemiparesis (PSOM > 0.5; mean age ± standard deviation [s.d.]: 15.49 ± 4.64 years), while nine had a good clinical outcome (PSOM = 0; mean age ± s.d.: 15.24 ± 4.10 years). 18 healthy subjects (mean age ± s.d.: 15.39 ± 4.28 years) were matched regarding age and gender.

Significant group differences occurred in upper limb function measured with HSS, ULMQS, and the ABILHAND questionnaire (Table 1). Patient groups did not differ in terms of lesion side, lesion location, lesion volume, or cortical reorganization (*P* > 0.05; Table 1). Further, in typically developing peers and patients, TMS revealed a contralateral corticospinal tract wiring pattern. For more detailed information, see Supplementary Table S1 and S2 online.

**Table 1:** Participants’ characteristics and clinical outcome.

	Peers	Patients		Test	value	df	P
		Good clinical outcome	Hemiparesis				
	M ± s.d [range]	M ± s.d [range]	M ± s.d [range]				
N	18	9	9				
Age at assessment (years)	15.39 ± 4.28 [9.58–24.41]	15.24 ± 4.10 [9.5–22.66]	15.49 ± 4.64 [9.41–23.08]				
**Sex, n (%)**							
Female	6 (72.7)	3 (33.3)	3 (33.3)				
Male	12 (27.3)	6 (66.7)	6 (66.7)				
**Motor outcome**							
ABILHAND-Kids	6.68 ± 0.0 [6.68–6.68]	5.55 ± 1.21 [3.51–6.86]	3.87 ± 2.14 [1.03–6.86]	Kruskal–Wallis	22.47	2	0.000***
ULMQS	6.22 ± 8.33 [0.00–25.00]	14.61 ± 15.14 [0.00–47.62]	135.71 ± 101.5 [16.95–303.03]	Kruskal–Wallis	21.17	2	0.000***
HSS	51.81 ± 34.26 [10.2–177.8]	30.7 ± 23.34 [4.98–67.80]	184.18 ± 110.8 [18.76–309.09]	Kruskal–Wallis	13.19	2	0.001**
**Stroke characteristics**							
Age at stroke (years)		7.6 ± 5.95 [0.08–15.58]	6.16 ± 5.16 [0.08–14.66]	Mann–Whitney U	27.00	2	0.315
Time since stroke		7.53 ± 3.72 [3.58–15.50]	9.43 ± 2.6 [5.75–13.42]	Mann–Whitney U	56.00	2	0.173
**Lesion side, n (%)**							
Left		8 (88.9%)	7 (77.8%)	Chi-square	0.72	2	0.559
Right		1 (11.1%)	2 (22.3%)				
**Location, n (%)**							
Cortical		1 (11.1%)	1 (11.1%)	Chi-square	2.215	2	0.330
Subcortical		8 (88.9%)	7 (77.8%)				
Both		0 (00.0%)	1 (11.1%)				
Lesion size (ratio × 1000)^+^		0.74 ± 1.52 [0.0–3.79]	0.95 ± 0.98 [0.01–2.65]	Mann–Whitney U	52.00	2	0.315

*peers* typically developing peers, *HSS* total hand strength score, *ULMQS* Upper Limb Movement Quality Score.

Lesion size = lesion size/total intracranial volume.

Significant at: *P < 0.05; **P < 0.01; ***P < 0.001.

### Between-group differences in the motor network

**Figure 1:**
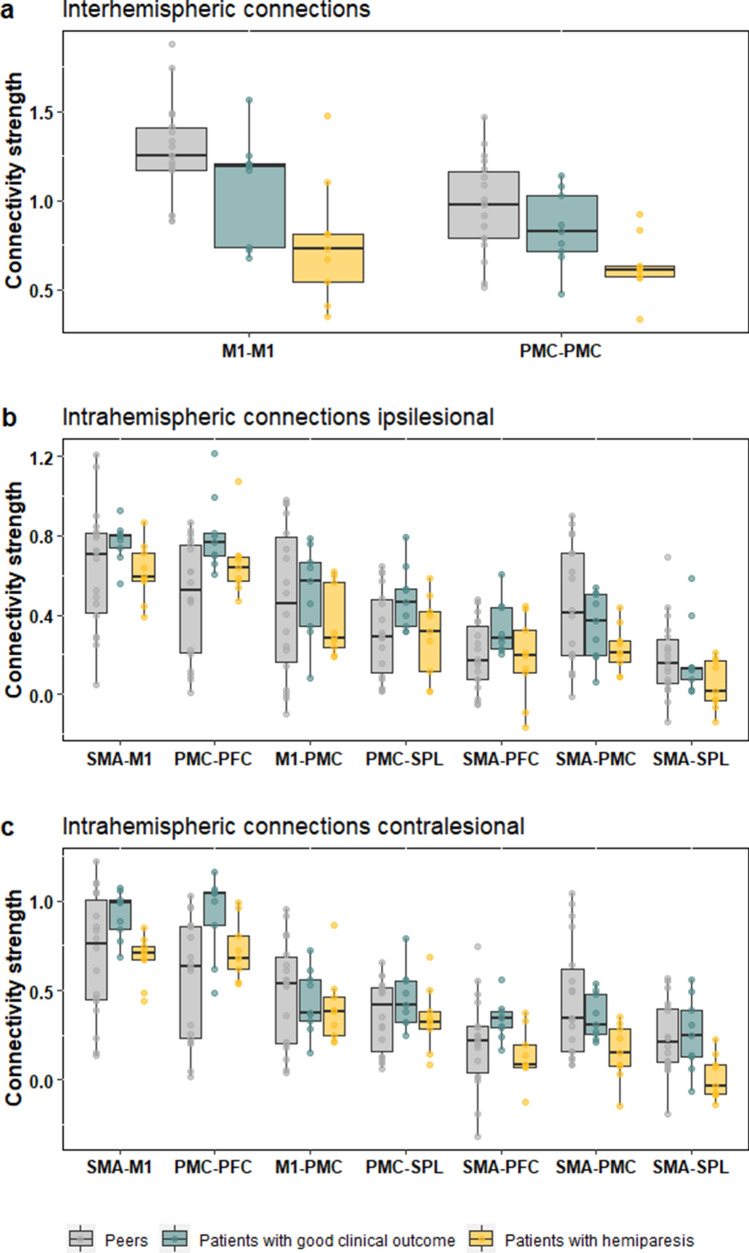
Comparison of connectivity strength. For typically developing peers (gray), patients with good clinical outcome (green), and patients with hemiparesis (yellow), the boxplots show the ROI-to-ROI connectivity strength within the motor network including the prefrontal cortex (PFC), dorsal premotor cortex (PMC), the primary motor cortex (M1), the supplementary motor area (SMA), and the superior parietal lobe (SPL).

Overall, all three groups had higher inter- than intrahemispheric connectivity strengths. Figure 1 depicts all inter- and intrahemispheric connectivity of the wider motor network, which was based on the model by Sharma and Cohen^32^.

#### Interhemispheric connectivity

Significant group differences occurred for the connectivity strength between the left and right primary motor cortex (M1–M1: H(2) = 11.78, *P* = 0.003, Fig. 1) and between the left and right dorsal premotor cortex (PMC–PMC: H(2) = 9.21, *P* = 0.010, Fig. 1). Post-hoc tests showed that M1–M1 and PMC–PMC connectivity strength was significantly lower in patients with hemiparesis compared with patients with good clinical outcome (PMC–PMC: H(2) = 17.00, *P* = 0.038, Fig. 1) and peers (M1–M1: H(2) = 18.00, *P* = 0.001; PMC–PMC: H(2) = 26.00, *P* = 0.005, Fig. 1). Patients with good clinical outcome and peers showed no significant difference for M1–M1 and PMC–PMC connectivity (M1–M1: H(2) = 49.00, *P* = 0.120; PMC–PMC: H(2) = 58.00, *P* = 0.237; Fig. 1).

#### Intrahemispheric connectivity

We found significant group differences for ipsilesional connectivity strength between PMC–PFC (H(2) = 8.75, *P* = 0.013, Fig. 1) and contralesional connectivity strength between PMC–PFC (H(2) = 8.75, *P* = 0.013, Fig. 1), SMA–PMC (H(2) = 6.08, *P* = 0.048, Fig. 1), SMA–PFC (H(2) = 6.42, P = 0.040, Fig. 1), and SMA–SPL (H(2) = 8.70, *P* = 0.013, Fig. 1). Post hoc tests showed that patients with hemiparesis had significantly lower ipsilesional and contralesional connectivity strength between PMC–PFC, SMA–PFC and SMA–SPL compared with patients with good clinical outcome (ipsilesional PMC–PFC: U(2) = 16.00, *P* = 0.031; contralesional PMC–PFC: U(2) = 17.00, *P* = 0.038; SMA–PFC: U(2) = 11.00, P = 0.008; SMA–SPL (U(2) = 13.00, *P* = 0.014; Fig. 1) and between contralsional SMA–SPL compared with peers (U(2) = 42.00, *P* = 0.045, Fig. 1). Further, patients with good clinical outcome had significantly higher intrahemispheric connectivity strength in ipsi- and contralesional PMC–PFC (ipsilsional: U(2) = 25.00, *P* = 0.017; contralesional: U(2) = 26.00, *P* = 0.004; Fig. 1) and ipsilesional PMC-SPL (U(2) = 40.00, *P* = 0.035, Fig. 1) compared with peers.

#### Average network connectivity

**Figure 2:**
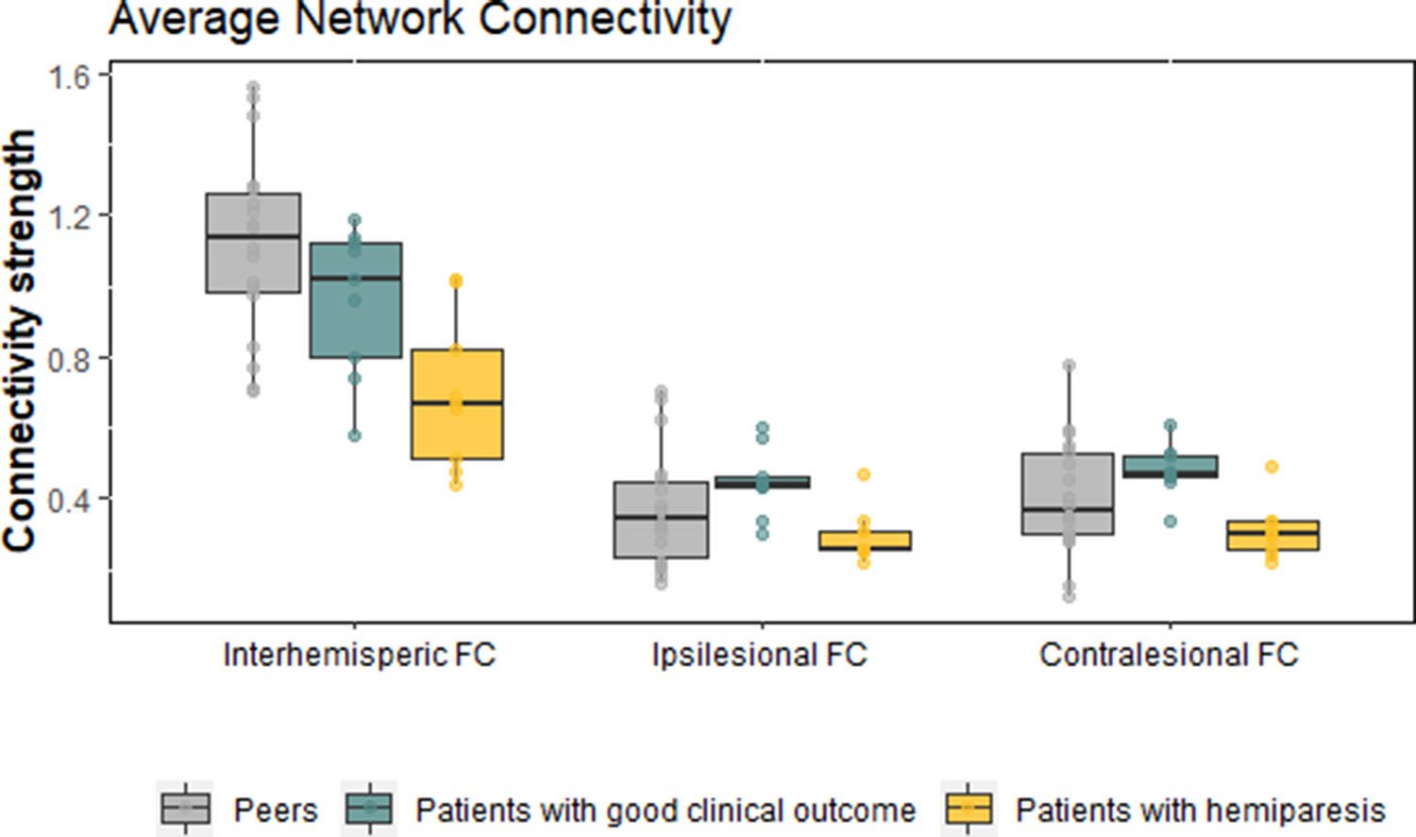
Between-group differences in motor network connectivity between patients with hemiparesis, patients with good clinical outcome, and typically developing peers. Patients with hemiparesis displayed significantly lower interhemispheric network connectivity than typically developing peers. Patients with good clinical outcome displayed significantly higher intrahemispheric network connectivity in the ipsilesional and contralesional hemisphere. Error bars show the standard error of the mean; (FC) = functional connectivity.

Related to our a-priori hypotheses on alterations in intra- and interhemispheric connectivity, we calculated the average network connectivity. Analyses of average inter- and intrahemispheric network connections revealed significant differences in average network connectivity between groups (interhemispheric connectivity: H(2) = 12.62, *P* = 0.002; intrahemispheric connectivity, contralesional: H(2) = 8.70, *P* = 0.013; intrahemispheric connectivity, ipsilesional: H(2) = 5.99, *P* = 0.050, Fig. 2).

Post hoc tests of interhemispheric connections showed that patients with hemiparesis had significantly lower connectivity strength compared with patients with good clinical outcome (U(2) = 15.00, *P* = 0.024) and peers (U(2) = 16.00, *P* = 0.001), while patients with good clinical outcome showed no significant difference in connectivity strength compared with peers (U(2) = 56, *P* = 0.605).

Post hoc tests of intrahemispheric connections showed that patients with good clinical outcome had significantly higher connectivity strength compared with patients with hemiparesis (contralesional, U(2) = 9.00, *P* = 0.004; ipsilesional, U(2) = 6.00, *P* = 0.001). Patients with hemiparesis showed no significant difference in connectivity strength compared with peers.

### Association between average network connectivity and asymmetry of upper limb function

**Figure 3:**
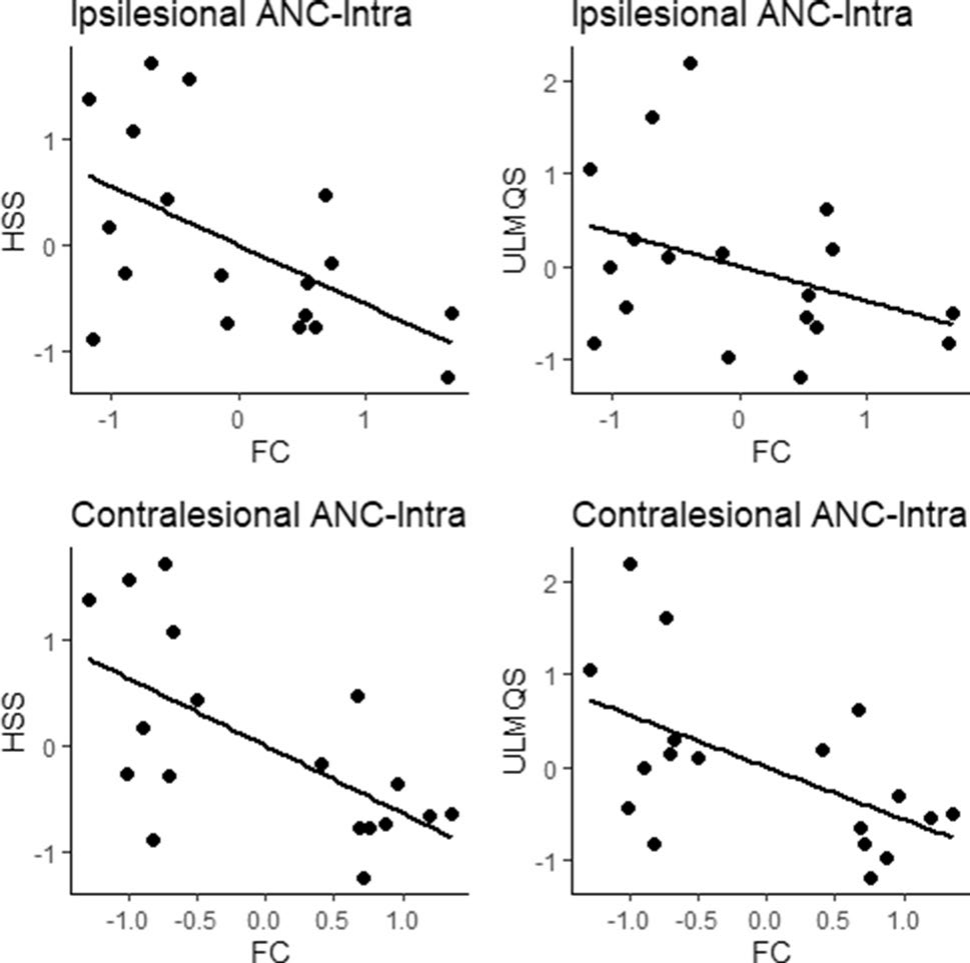
Associations between asymmetry of upper limb function, manual ability, and average network connectivity. Scatter plots depict residuals of the partial correlations between motor assessments (HSS, ULMQS and ABILHAND) and inter-/intrahemispheric average network connectivity with age at assessment, and age at stroke, lesion size as covariates. *ANC-intra* intrahemispheric average network connectivity, *FC* functional connectivity, *HSS* hand strength score, *ULMQS* Upper Limb Movement Quality Score.

To investigate the relationship between average network connectivity, asymmetry of upper limb function, and manual ability, we performed partial correlation analyses with age at assessment, age at stroke, and lesion size as covariates. We found that average intrahemispheric connectivity was inversely related to asymmetry between the left and right hand in the HSS (contralesional: *r* = 0.63, *P* = 0.011; ipsilesional: *r* = 0.54, *P* = 0.029, Fig. 3) and ULMQS (contralesional: *r* = 0.54, *P* = 0.034, Fig. 3). None of the other correlations was statistically significant (*P* > 0.05).

## Discussion

This cross-sectional study used resting-state fMRI and TMS to investigate mechanisms of motor recovery in patients after pediatric AIS with hemiparesis, patients after pediatric AIS with good motor outcome, and typically developing peers. Patients as well as peers had a contralateral corticospinal tract wiring as expected in a typically developing brain. As hypothesized, we found that patients with hemiparesis had lower interhemispheric connectivity strength, particularly between the primary motor cortices, compared with patients with good clinical outcome and peers. Interestingly, patients with good motor outcome had similar *inter*hemispheric connectivity strength as peers, but even higher *intra*hemispheric connectivity strength (particularly in the unaffected hemisphere) than peers. These results are in agreement with the work of Cramer, et al.^33^ and Mintzopoulos, et al.^30^ showing enhanced intrahemispheric connectivity in successful motor recovery. Further, confirming our second hypothesis, we found that higher asymmetry of upper limb function was associated with lower average intrahemispheric connectivity, both contra- and ipsilesional.

Our finding of reduced interhemispheric connectivity, especially between the motor cortices, in patients with hemiparesis is in line with findings from adult stroke patients^21,26,27,34–36^. The reduction of connectivity strength between the primary motor cortices even after more than two years post-stroke suggests that interhemispheric connectivity has not yet recovered. This in turn seems to promote the persistence of hemiparesis. In patients who successfully recovered, connectivity strength between the primary motor cortices was comparable to typically developing peers. The findings reaffirm the importance of interhemispheric connections in upper limb function and recovery, and show the valuable insight into adaptive (re)organization following early brain injury that can be gained from the assessment of a cohort of patients with good clinical outcome after AIS. Overall, these findings highlight the relevance of the integrity of interhemispheric connectivity for motor function and provide further support that assessment of restoration and normalization of altered interhemispheric connectivity measured with rs-fMRI is a reliable index for evaluating the effectiveness of rehabilitation at the neural level^21,29,36^.

Regarding intrahemispheric connections, we found lower ipsi- and contralesioinal connectivity strength between the SMA and the superior parietal lobe (SMA-SPL) and the prefrontal cortex (SMA-PFC) as well as between the dorsal premotor cortex and the prefrontal cortex (PMC-PFC) in patients with hemiparesis compared with patients with good clinical outcome. In comparisons with peers, the contralesional connectivity strength between the SMA and superior parietal lobe (SMA-SPL) was lower in patients with hemiparesis. In contrast, patients with good motor outcome had higher intrahemispheric connectivity strength in the ipsi- and contralesional hemisphere compared with peers. Increased interlinking of motor regions in the contralesional hemisphere might be beneficial for motor recovery and indicative for an adaptive neuronal response that facilitates reorganization. In line with this assumption, successful motor recovery in the chronic phase has been associated with higher intrahemispheric connectivity between M1-SMA^30^ as well as increased activation in the SMA during a finger tapping task^33^. The between-group differences in intrahemispheric connections (SMA–PFC, and SMA–SPL) are in line with previous findings that suggest a crucial role of the SMA for upper limb functioning^37,38^. The SMA seems to be involved early on in the process of stroke recovery^37,38^ and has increased effective connectivity in chronic stroke patients performing motor imagery tasks^26,30,32^. Indeed, increased SMA activity (i.e., by high-frequency transcranial magnetic or direct current stimulation) has been suggested as a potential means for ameliorating M1 dysfunction after stroke^39,40^.

The assessment of the relationship between average network connectivity and motor outcome showed that average network connectivity was associated with asymmetry of upper limb function, as hypothesized. The stronger association between intrahemispheric network connectivity (as opposed to interhemispheric connectivity) with asymmetry of upper limb function might be due to the particular importance of the contralesional hemisphere for motor function recovery, especially after early unilateral brain injury^41,42^.

A possible way to interfere with and ultimately alter network connectivity is by means of non-invasive brain stimulation that intervenes at the neural level^43,44^ For instance, motor outcome was improved after intermittent theta-burst stimulation over the ipsilesional primary motor cortex after acute stroke compared with stimulation over the control region, the parieto-occipital vertex^44^. This suggests that brain stimulation can improve recovery of functional connectivity in the motor network and promote good motor outcome. Similarly, transcranial direct current stimulation was found to induce significant improvement of motor functions and higher functional connectivity strength between ipsilesional M1 to contralateral PMC and between bilateral precuneus in patients after stroke^43^. Yet, this effect has to be replicated in children after AIS.

This study had some strengths that merit comment. First, the testing protocol consisted of state-of-the-art clinical assessments for motor function, an rs-fMRI scan, and TMS. Second, an extended and standardized test battery was adopted to investigate several motor modalities of the body structure, the body function, and the activity domain of the International Classification of Functioning, Disability and Health framework^45^. Third, as our study included children with childhood and neonatal AIS, TMS was used to ensure that only patients with contralateral reorganization were considered. Thus, an influence of different cortical reorganizations on connectivity can be excluded. Fourth, not only the affected upper limb of the patients was assessed, but also the contralateral upper limb. Lastly, a patient group with good clinical outcome after AIS (no neurological deficits; PSOM = 0) was included as an additional control group to the group of typically developing peers. Our findings emphasize the importance of including patients with good clinical outcome in the analyses to help disentangle different reorganization patterns in relation to motor function; a practice that—to our knowledge—has not been adopted so far.

Nevertheless, our study also has some limitations. First, the effect of the lesion area on the connectivity data is uncertain. To date, various approaches have been adopted to correct for the potential bias introduced by the lesion area. One approach is to exclude the lesion area during spatial normalization^46^, while another is to exclude the ROIs that overlap with the lesion area^11,47^. However, in our study sample none of the patients with lesions involving the cortex (n = 3) had overlaps with our predefined ROIs in the motor network. Second, our sample size was relatively small. The incidence of pediatric AIS is low with 2.1/100,000 children and 13 neonates per 100,000 live births, which reduces the number of patients available for possible recruitment. Therefore, further research is needed to replicate our findings in a larger cohort and confirm that brain connectivity in the motor network is related to motor function throughout post-stroke recovery. Third, the study sample included children across a wide age range at time of assessment. This is a considerable limitation considering the different neurodevelopmental stages of the patients. Yet, the rareness of the event with one to two cases per center per year in Switzerland based on estimates from the SNPSR from 2000 to 2019 makes recruitment a challenging task. Thus, we tried to adjust for this by matching the control and patient group by age and gender and by including age at assessment as a covariate into the analysis. Finally, future studies should examine the extent to which rehabilitation efforts can increase inter- and intrahemispheric connectivity strength of the motor network.

In this study, we found that patients with hemiparesis have lower interhemispheric connectivity, while patients with a good clinical outcome have higher intrahemispheric connectivity strength in the unaffected hemisphere. Higher intrahemispheric functional network connectivity was related to asymmetry of motor function. This could have important clinical and theoretical implications. First, functional connectivity measurements of resting-state activity might be a predictor for motor impairment after stroke. Second, changes in resting-state functional connectivity may be used to track the process of recovery longitudinally, in particular as a marker of neuronal recovery. Identification of optimal treatment strategies to improve recovery is still limited by the large variance in outcomes of patients after AIS. Thus, identifying biomarkers that distinguish patient subgroups will help to elucidate factors that are important for successful recovery after pediatric AIS. Overall, the association between motor functions and resting-state functional connectivity suggests that intrinsic brain activity may represent a biomarker for tracking rehabilitation.

## Methods

### Participants

Participants were recruited from 2014 through 2017 as part of the Hemispheric Reorganisation (HERO) study^48^, approved by the Research Ethics Committee of Bern, Switzerland. Patients were identified by the Swiss Neuropaediatric Stroke Registry (SNPSR)—a multicenter, prospective, and population-based registry that includes children diagnosed with AIS under the age of 16 years^1^. Patients met the following inclusion criteria: Diagnosis of AIS (confirmed by MRI or computed tomography) before the age of 16 years and at least two years prior to recruitment in the chronic phase, and older than five years of age to enable adequate compliance, and contralateral corticospinal tract wiring. For more detailed information see the previously published study protocol^48^. The control group, a sample of typically developing peers comparable in age and sex to the patients’ groups, was recruited through advertisements on the hospital intranet and flyers. Participants were excluded if they had neurological disorders unrelated to AIS, ferrous implants, active epilepsy, claustrophobia, developmental delay, or behavioral problems that could affect ability to comply with study requirements (see Supplementary Fig. S1).

Of the 379 patients identified from the SNPSR who met the inclusion criteria, 96 were not contacted: 20 had died, 7 had either trisomy 21, epilepsy, other severe handicaps, or severe behavioral problems, 12 were living abroad, and for 57 consent for SNPSR or follow-up studies was lacking. Of the remaining 283 patients who were contacted personally by mail and subsequently by phone, 120 did not respond and 135 reported a lack of motivation or felt that the duration and type of assessment (MRI, TMS) would be inconvenient. Of the remaining 28 patients, 2 had to be excluded because of developmental delay or behavioral problems interfering with compliance with the test conditions, 2 had an erroneous fMRI sequence, and 2 retainer artefacts. Another 4 were excluded because of the lesion being bilateral. Thus, the final sample consisted of 18 patients diagnosed with chronic AIS (see Supplementary Fig. 1). We matched the control group of typically developing peers using a 1:1 ratio by age and gender (n = 18).

All participants, or their parent or guardian if they were younger than 18 years, gave written informed consent, according to the Code of Ethics of the World Medical Association (Declaration of Helsinki).

### Clinical outcome assessment

All participants underwent a standardized neurological examination performed by a research physician (J.D and S.G) at the Children’s University Hospital, Inselspital, Bern, Switzerland. Details of data collection and study design have been previously reported^48^. To study motor outcome, an extended and standardized test battery was adopted to investigate several domains as proposed by the International Classification of Functioning, Disability and Health framework^45^, including body structure (anatomical structure of the body), and body function (physiological function of the body), activity (execution of a task or action), and participation (involvement in everyday life situations).

#### Disease-specific outcome

The Pediatric Stroke Outcome Measure (PSOM)^49^ was used to measure disease-specific outcome at the time of MRI scanning. The PSOM assesses neurological deficits and consists of five subscales for right and left sensorimotor functioning, language production, language comprehension, and cognition/behavior. We used the sensorimotor subscale to classify the presence of hemiparesis (0 = no sensorimotor deficit; 0.5 = mild deficit, with normal function; 1 = moderate deficit, with decreased function; 2 = severe deficit with no function). Patients with scores greater than 0.5 on the sensorimotor subscale were classified as having hemiparesis^50,51^. Patients with a score of zero on all subscales were classified as having a good clinical outcome. Detailed information on each participant is provided in Supplementary Table S2.

#### Upper limb function

**Hand strength.** Palmar grasp strength and thumb–forefinger pinch strength were measured with a dynamometer (30 Psi pneumatic dynamometer Baseline, USA and a 30 lb mechanical pinch gauge, Baseline, USA). Participants were instructed to perform each task with maximal effort (repeating it three times with a 30-s break between attempts). After performing the task with each hand separately, the maximum values for both hands were averaged to yield a total hand strength score (HSS).

**Quality of upper limb function.** Quality of upper limb function was assessed using the Melbourne Assessment of Unilateral Upper Limb Function, version 2^52,53^. This test contains 14 tasks (e.g. grasping and releasing, manipulating, reaching) that cover four basic upper limb functions, namely, range of movement, accuracy of reaching and pointing, dexterity of reaching and manipulating, and fluency of movement. The assessment was performed for both the left and the right side. The values of all items were summarized to yield a total Upper Limb Movement Quality Score (ULMQS) for each side.

**Asymmetry of upper limb function.** Using the assessment of HSS and ULMQS for both upper limbs allowed us to calculate the asymmetry between the dominant and non-dominant hand^51^ with the following Eq. (1):

1 $$Asymmetry index=\frac{scor{e}_{dominant side}-scor{e}_{nondominant side }}{scor{e}_{dominant side}+scor{e}_{nondominant side}}100$$

An asymmetry index of zero represents perfect symmetry between the dominant and non-dominant side, whereas an index larger than zero represents asymmetry towards the dominant side.

#### Manual ability in everyday life

Manual ability in everyday life situations was assessed using the ABILHAND-Kids questionnaire^54^. This is a parent-reported outcome measure assessing the use of the upper limbs in everyday situations (0 = impossible, 1 = difficult, or 2 = easy to perform). The total score ranges from + 6.68 (all items easy to perform) to − 6.75 (all items impossible to perform).

#### Cortical Reorganization

Transcranial magnetic stimulation (TMS) was performed to determine the type of cortical reorganization after AIS^11^. For this purpose, silver-silver chloride surface electrodes (APLINE, bioMEd) were mounted in a tendon-belly arrangement over the Abductor Pollicis muscle on both hands^55^. A Neurodata amplifier system connected to an IPS230 Isolated Power System (Grass-Telefactor, Braintree, MA, USA) was used for pre-amplification (1000x) and as bandpass filter (10–1000 Hz) of the EMG signals. The inputs were entered into a computer-assisted data acquisition system (sampling rate 5 kHz)^56^. The EMG signal peak-to-peak amplitudes were calculated for all derived muscles in a 65 ms time window. Single-pulse monophasic TMS pulses were delivered over both hemispheres. Both hemispheres were examined according to the stimulation response in the contralateral and/or ipsilateral upper extremity.

Cortical reorganization was defined according to the stimulation response in the ipsilateral or contralateral Abductor Pollicis brevis muscle^11^: a stimulation response only in the contralateral Abductor Pollicis Brevis muscle were defined as *contralateral reorganized*, stimulation response only in the ipsilateral Abductor Pollicis Brevis muscle was defined as *ipsilateral reorganized*, and stimulation response in both the ipsilateral and contralateral Pollicis Brevis muscle was defined as *mixed*. The methodology was carried out in accordance with the safety regulations and guidelines^57^ and is described in detail in the Supplementary Material.

### Neuroimaging

The MRI protocol was carried out in accordance with the safety regulations and guidelines^48,58^. All MRI recordings were performed on a 3 T scanner (Magnetom Verio, Siemens, Erlangen, Germany) equipped with a 32-channel phased-array head coil at the Inselspital, Bern University Hospital, Switzerland.

#### Structural MRI data

High-resolution anatomical T1-weighted images were acquired with a magnetization-prepared rapid acquisition gradient-echo (MP-RAGE) sequence with the following parameters: repetition time (TR) = 2530 ms; echo time (TE) = 2.92 ms; inversion time (Ti) = 1100 ms; flip angle (FA) = 9°; field-of-view (FOV) = 256 mm × 256 mm; matrix dimension = 256 × 256; isotropic voxel resolution = 1 mm^3^; with a total of 160 sagittal slices.

Lesion characteristics such as location, size, and side affected were obtained from anatomical images (T1) by a board-certified neuroradiologist (N.S.). Lesions were classified according to the hemisphere affected (left, right, or bilateral) and anatomical location (cortical, subcortical, or both cortical and subcortical). To calculate the volume of affected brain tissue in the chronic stage, ischemic lesions were manually traced on T1 weighted images acquired the same day as functional imaging. Lesion size was defined as the affected brain tissue in relation to the total brain volume (lesion volume [cm^3^]/total brain volume [cm^3^]). Total brain volume was calculated using the statistical parametric mapping toolbox (SPM12, Wellcome Department of Imaging Neuroscience, London, England).

#### Functional MRI data

The BOLD rs-fMRI images were recorded with a multiband echo planar imaging T2*-weighted sequence “mb-EPI” (Feinberg et al., 2013) and had the following parameters: TR = 300 ms; TE = 30 ms; FA = 90°; FOV = 230 × 230 mm; pixel size = 3.6 × 3.6 mm; matrix dimension = 64 × 64; 32 slices positioned in the line between the anterior and posterior commissure (interleaved ascending acquisition order); slice thickness = 3.6 mm; isotropic voxel resolution of 3.6 mm^3^; and a total of 1000 images were recorded. The fMRI time-series were acquired with a 2 GRAPPA acceleration factor and a 3D prospective acquisition correction mode.

Data was pre-processed using the functional connectivity toolbox (CONN, version 17)^59^ as implemented on the platform MATLAB (R2017; MathWorks, Natick, MA, USA). We used the standard preprocessing pipeline^59^. First, functional images from patients with lesions in the right hemisphere (n = 3) were flipped along the midsagittal plane, so that the affected hemisphere corresponded to the left hemisphere in the whole patient sample. Second, by visual inspection, we verified that none of the patients with lesions involving the cortex (n = 3) had overlaps with our predefined ROIs in the motor network. There were no cortical lesions overlapping with regions included in the analysis. Third, the structural images were segmented to allow creation of white matter and cerebrospinal fluid (CSF) masks. The spatial preprocessing of the functional images included the correction of slice time, realignment, normalization, and smoothing (applying the Gaussian filter kernel, FWH = 8 mm). Quality of registration and parcellation was assessed by visual inspection of each subjects’ data. Fourth, the temporal processing of the functional images took into account potential confounding factors, such as movement parameters and artifacts. BOLD signals obtained from white matter and CSF masks were also included. All these temporal confounding factors were regressed out from the functional images using a generalized linear model framework. Finally, the functional images were filtered using a “band-pass filter” (0.01–0.1 Hz).

**Figure 4:**
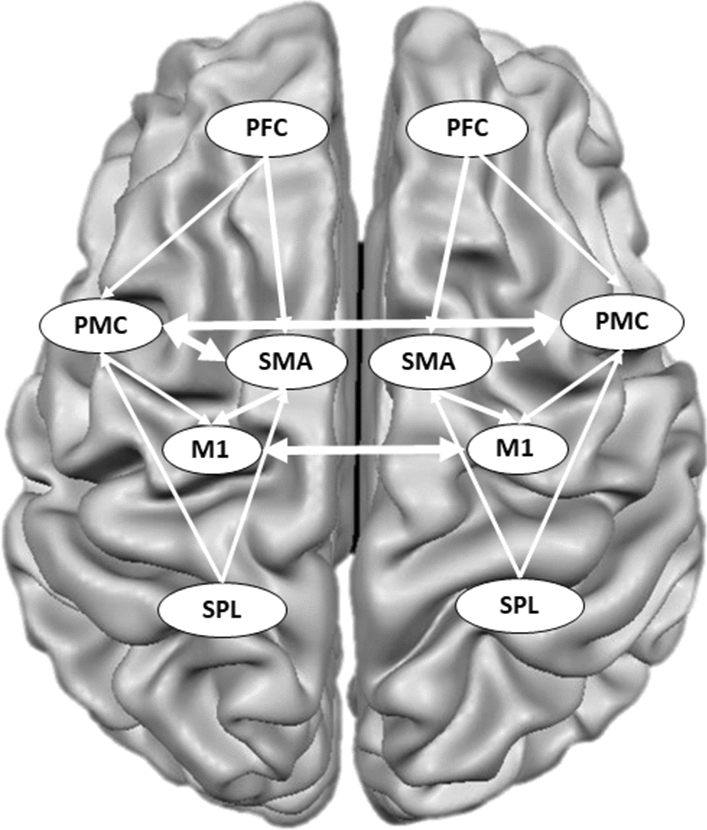
The regions of interest (ROI) of the motor network. The functional connectivity analyses included the following ROIs: prefrontal cortex (PFC), dorsal premotor cortex (PMC), primary motor cortex (M1), supplementary motor area (SMA), superior parietal lobe (SPL) (adapted from Sharma et al.^32^). Altogether, this motor network consists of 14 intrahemispheric and 2 interhemispheric connections.

Functional connectivity was assessed in predefined ROIs of an extended model of the motor network (Fig. 4). This extended model was based on the previous literature on stroke recovery in humans and animals^32^ and included the bilateral areas of M1, prefrontal cortex (PFC), dorsal premotor cortex (PMC), supplementary motor area (SMA), and superior parietal lobe (SPL). Together, these five ROIs represent a motor network with 14 intrahemispheric and two interhemispheric connections (Fig. 4). For data extraction from those ROIs, we used the brain parcellation atlas from the CONN toolbox^59^. For each participant, we extracted the mean time-series by averaging across all voxels in each ROI and computed bivariate correlation coefficients for each pair of ROIs. For further analyses, we Fisher z-transformed the correlation coefficients.

Related to our hypotheses on alterations in intra- and interhemispheric connectivity, we calculated the indices of average network connectivity: (1) ROI-to-ROI correlation coefficients of all connections in the ipsilesional (7 connections) and contralesional hemisphere (7 connections) were averaged for each participant to obtain average intrahemispheric network connectivity of the ipsilesional and contralesional hemisphere, (2) ROI-to-ROI correlation coefficients between homologous regions (e.g. M1–M1 and PMC–PMC; two connections) were averaged for each participant to obtain average interhemispheric network connectivity.

### Statistical analyses

To test our primary hypothesis that patients after AIS with hemiparesis have lower inter- and intrahemispheric functional connectivity compared with patients with good motor outcome and typically developing peers, we used the non-parametric Kruskal–Wallis test, and the Mann–Whitney U-test for post hoc pairwise comparison.

To test our secondary hypothesis that asymmetry of upper limb function and manual ability (assessed by HSS, ULMQS, and ABILHAND-Kids) is related to average inter- and intrahemispheric network connectivity, we used partial Spearman correlation analyses with age at assessment, age at stroke, and lesion size as covariates. For visualization of the results, we extracted the partial correlations’ residuals.

All analyses were performed using the statistical software package R 3.6.0^60^. To account for the effects of multiple hypothesis testing (type I error), false discovery rate (FDR) correction was employed. Results of *P* < 0.05 FDR-corrected were considered significant.

## Supplementary Information

Supplementary Information

## Data Availability

The datasets generated and analyzed during this study are available from the corresponding author on reasonable request.
