# Supplementary material for: Functional connectivity and upper limb function in patients after pediatric arterial ischemic stroke with contralateral corticospinal tract wiring
Source: Sci Rep. 2021 Mar 9;11:5490. doi: 10.1038/s41598-021-84671-2 (PMC7943570; doi:10.1038/s41598-021-84671-2)
Supplement: Supplementary file 1 — Supplementary Information [file 41598_2021_84671_MOESM1_ESM.docx]

**Supplementary Material for**

**Functional connectivity and upper limb function in patients after pediatric arterial ischemic stroke with contralateral corticospinal tract wiring**

Leonie Steiner, Stephanie Homan, Regula Everts, Andrea Federspiel, Sandeep Kamal, Juan Delgado Rodriguez, Salome Kornfeld, Nedelina Slavova, Roland Wiest, Alain Kaelin, Maja Steinlin, Sebastian Grunt

**This file includes:**

**Supplementary Figure 1**

**Supplementary Table 1 & 2**

**Supplementary information for TMS**

| **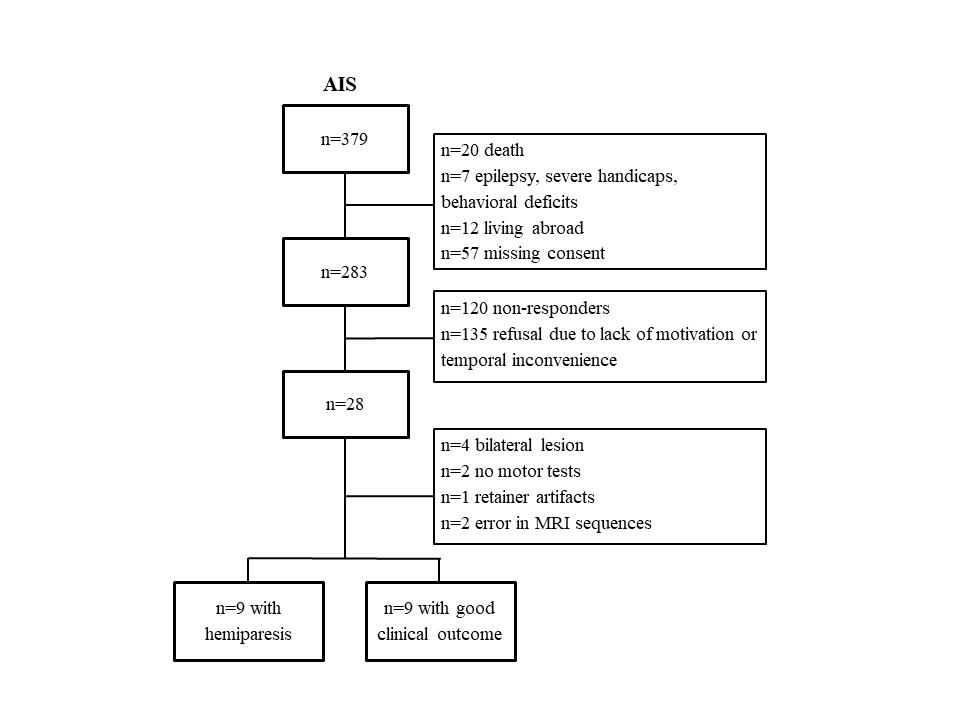** |
| --- |
| **FIGURE S1. Study population flowchart.** Of 379 patients from the Swiss Neuropaediatric Stroke Registry who met inclusion criteria, 96 were not contacted due to the following reasons: death (n =20), trisomy 21, epilepsy, other severe handicaps or heavy behavioral problems (n = 7), living abroad (n =12), missing consent for Swiss Neuropaediatric Stroke Registry or follow-up studies (n = 57). All 283 remaining patients were contacted by letter post and additionally by phone two weeks later if no answer was received. Of the 283 patients contacted, 120 were non-responders and 135 reported a lack of motivation or that the duration and type of assessment (MRI, behavioral assessments) would be inconvenient. A final sample of 28 patients agreed to participate and was examined. The typically developing control sample was recruited through posted advertisements around the University Children’s Hospital of Berne, Switzerland. AIS = patients with pediatric arterial ischemic stroke; n = number of participants. |

**Table S1.** Characteristics for individual participants.

| N | Group | Sex | Age at assessment  (years) | Handedness | Stroke | | | | | |
| --- | --- | --- | --- | --- | --- | --- | --- | --- | --- | --- |
|  |  |  |  |  | Age at stroke | Time since stroke | Lesion Side | Lesion Location | Lesion  size | CST wiring pattern |
|  |  |  |  |  | (years) | (years) |  |  |  |  |
| 1 | No hemi | M | 22.8 | Right | 14.6 | 8.2 | Left | SC | 3.79 | Contra |
| 2 | No hemi | F | 15.6 | Right | 11.9 | 3.8 | Right | SC | 0.01 | Contra |
| 3 | No hemi | F | 15.4 | Right | 7.1 | 7.5 | Left | SC | 0.02 | Contra |
| 4 | No hemi | M | 13.2 | Right | 5.9 | 7.4 | Left | SC | 3.47 | Contra |
| 5 | No hemi | M | 18.9 | Right | 14.4 | 4.5 | Left | SC | 0.05 | Contra |
| 6 | No hemi | M | 11.8 | Right | 6.8 | 4.1 | Left | SC | 0.01 | Contra |
| 7 | No hemi | F | 18.6 | Right | 14.8 | 3.9 | Left | SC | 0.01 | Contra |
| 8 | No hemi | M | 9.6 | Right | neonatal | 9.6 | Left | C | 0.02 | Contra |
| 9 | No hemi | M | 16.9 | Left | 1.2 | 15.6 | Left | SC | 0.09 | Contra |
| 1 | Hemi | M | 10.9 | Left | neonatal | 10.1 | Left | C | 0.02 | Contra |
| 2 | Hemi | F | 20.1 | Right | 10.5 | 10.5 | Left | SC | 0.01 | Contra |
| 3 | Hemi | M | 18.5 | Left | 6.3 | 12.1 | Left | SC | 1.08 | Contra |
| 4 | Hemi | F | 13.1 | Right | 3.5 | 10.6 | Right | SC | 0.35 | Contra |
| 5 | Hemi | M | 23.1 | Right | 15.7 | 7.6 | Left | SC | 2.12 | Contra |
| 6 | Hemi | M | 11.5 | Left | 3.6 | 7.1 | Left | SC | 0.01 | Contra |
| 7 | Hemi | M | 14.6 | Right | 8.8 | 5.9 | Right | SC + C | 0.41 | Contra |
| 8 | Hemi | M | 9.5 | Left | 1.6 | 7.1 | Left | SC | 1.00 | Contra |
| 9 | Hemi | F | 13.5 | Left | neonatal | 13.5 | Left | SC | 2.65 | Contra |

*Notes*. C, cortical; CST, corticospinal tract; F, female; Hemi, hemiparesis; M, male; SC, subcortical.

Calculation of lesion size ratio: volume of lesion/total intracranial volume × 1000.

|  | Group | Sensorimotor deficit | | Language production deficit | Language comprehension deficit | Cognitive or behavior deficit | Total  (0-10) |
| --- | --- | --- | --- | --- | --- | --- | --- |
| N |  | right | left |  |  |  |  |
| 1 | No hemi | 0 | 0 | 0 | 0 | 0 | 0 |
| 2 | No hemi | 0 | 0 | 0 | 0 | 0 | 0 |
| 3 | No hemi | 0 | 0 | 0 | 0 | 0 | 0 |
| 4 | No hemi | 0 | 0 | 0 | 0 | 0 | 0 |
| 5 | No hemi | 0 | 0 | 0 | 0 | 0 | 0 |
| 6 | No hemi | 0 | 0 | 0 | 0 | 0 | 0 |
| 7 | No hemi | 0 | 0 | 0 | 0 | 0 | 0 |
| 8 | No hemi | 0 | 0 | 0 | 0 | 0 | 0 |
| 9 | No hemi | 0 | 0 | 0 | 0 | 0 | 0 |
| 10 | Hemi | 1 | 0 | 0 | 0 | 0 | 1 |
| 11 | Hemi | 0.5 | 0 | 0 | 0 | 0 | 0.5 |
| 12 | Hemi | 1 | 0 | 0 | 0 | 0 | 1 |
| 13 | Hemi | 0 | 1 | 0 | 0 | 0 | 1 |
| 14 | Hemi | 0.5 | 0 | 0 | 0 | 0 | 0.5 |
| 15 | Hemi | 0.5 | 0 | 0 | 0 | 0 | 0.5 |
| 16 | Hemi | 0 | 1 | 0 | 0 | 0.5 | 1 |
| 17 | Hemi | 0.5 | 0 | 0 | 0 | 0 | 0.5 |
| 18 | Hemi | 1 | 0 | 0 | 0 | 0 | 1 |

**Table S2.** Pediatric stroke outcome measure (PSOM)

*Notes*. Hemi, hemiparesis; CST, corticospinal tract

# **Transcranial Magnetic Stimulation**

In stroke patients, we first stimulated the affected and then the unaffected hemisphere. In healthy controls, we first stimulated the hemisphere representing the dominant hand and then the hemisphere representing the non-dominant hand. For stimulation with monophasic pulses, we used a figure-of-8 coil (diameter 5cm, maximal field strength 2.89T), which was connected to a Magstim 200 (Magstim Company Limited, Whitland, UK). The coil was hand-held and placed tangentially to the scalp (5 cm lateral and 1 cm anterior of the vertex) with the intersection of both wings at an angle of 45° with the midline, to induce electrical current in the cortex in the posterior-anterior direction according to our standard protocol.^1,2^ The hotspot was first searched and was defined as the position in which a reproducible muscle response can be triggered with minimal stimulation intensity.^2^ Once the hotspot was localized, the resting motor threshold (rMT), i.e. the minimum stimulation intensity with which 5 out of 10 motor evoked potentials above 50 µV can be obtained, was defined. Twenty stimulations with 120 % rMT were applied with an interstimulus interval randomly varying between 5 and 7 sec^3^. The Abductor Pollicis Brevis muscle was first examined in a relaxed state as controlled by EMG activity^3^. If no motor evoked potentials could be generated, the examination was repeated with a facilitation (the patients were asked to squeeze a foam rubber ball slightly). If no ipsilateral or contralateral motor evoked potentials could be triggered in the pre-contracted muscle, the stimulation output was increased to 100% (or as high as the patient tolerated). Level of attention was checked by another investigator and if the subject closed their eyes, he was asked to open it.

**References**

1 Brasil-Neto, J. P. *et al.* Optimal focal transcranial magnetic activation of the human motor cortex: effects of coil orientation, shape of the induced current pulse, and stimulus intensity. *Journal of clinical neurophysiology : official publication of the American Electroencephalographic Society* **9**, 132-136 (1992).

2 Conforto, A. B., Z'Graggen, W. J., Kohl, A. S., Rosler, K. M. & Kaelin-Lang, A. Impact of coil position and electrophysiological monitoring on determination of motor thresholds to transcranial magnetic stimulation. *Clin Neurophysiol* **115**, 812-819, doi:10.1016/j.clinph.2003.11.010 (2004).

3 Kaelin-Lang, A. & Cohen, L. G. Enhancing the quality of studies using transcranial magnetic and electrical stimulation with a new computer-controlled system. *Journal of Neuroscience Methods* **102**, 81–89 (2000).

Figure Legends

**FIGURE S1. Study population flowchart.** Of 379 patients from the Swiss Neuropaediatric Stroke Registry who met inclusion criteria, 96 were not contacted due to the following reasons: death (n =20), trisomy 21, epilepsy, other severe handicaps or heavy behavioral problems (n = 7), living abroad (n =12), missing consent for Swiss Neuropaediatric Stroke Registry or follow-up studies (n = 57). All 283 remaining patients were contacted by letter post and additionally by phone two weeks later if no answer was received. Of the 283 patients contacted, 120 were non-responders and 135 reported a lack of motivation or that the duration and type of assessment (MRI, behavioral assessments) would be inconvenient. A final sample of 28 patients agreed to participate and was examined. The typically developing control sample was recruited through posted advertisements around the University Children’s Hospital of Berne, Switzerland. AIS = patients with pediatric arterial ischemic stroke; n = number of participants.
